# Digitale Kompetenzen im Medizinstudium: Ergebnisse einer interdisziplinären Lehrveranstaltung

**DOI:** 10.1007/s00106-023-01411-w

**Published:** 2024-01-24

**Authors:** Anna-Maria Waibel, Martina Bischoff

**Affiliations:** 1 Studiendekanat, Medizinische Fakultät, Albert-Ludwigs-Universität Freiburg, Breisacher Str. 153, 79110 Freiburg im Breisgau, Deutschland; 2 Institut für Allgemeinmedizin, Universitätsklinikum Freiburg, Freiburg im Breisgau, Deutschland

**Keywords:** Medizinstudierende, E‑Health, Blended Learning, Telemedizin, Künstliche Intelligenz, Medical students, Digital health, Blended learning, Telemedicine, Artificial intelligence

## Abstract

**Hintergrund:**

Digitale Medizin erlangt zunehmend an Bedeutung, insbesondere durch den verstärkten Einsatz von künstlicher Intelligenz (KI) und Telemedizin. Die COVID-19-Pandemie hat diesen Trend beschleunigt und die Notwendigkeit digitaler Kompetenzen im Gesundheitswesen betont. Digitale Kompetenzen werden bisher im Medizinstudium jedoch unzureichend vermittelt.

**Ziel:**

Durch die Einführung eines Wahlfachs für digitale Kompetenzen in der Medizin wird eine curriculare Lücke an der Medizinischen Fakultät der Universität Freiburg geschlossen.

**Methoden:**

Das Wahlfach wurde von einer interdisziplinären Arbeitsgemeinschaft entwickelt und umfasst 7 Module zu Themen wie E‑Health, Telemedizin, KI, Krankenhausinformationssystemen und Data Literacy. Jedes Modul besteht aus einer vorbereitenden Selbstlerneinheit, einem theoretischen Teil und einem praktischen Teil. Eine Evaluation wurde durchgeführt, um die Wirksamkeit des Wahlfachs zu bewerten.

**Ergebnisse:**

Im Sommersemester 2022 nahmen 6 Studierende am Wahlfach teil. Die Evaluation zeigt, dass die Ziele des Wahlfachs erreicht wurden. Die Teilnehmenden konnten theoretisches Wissen erwerben und dieses durch praktische Übungen vertiefen.

**Schlussfolgerung:**

Die interdisziplinäre Gestaltung des Wahlfachs fördert den Austausch zwischen Studierenden und Lehrenden und schafft Synergien. Das modulare Format des Wahlfachs ermöglicht es, auch aktuelle Themen, wie generative KI, aufzugreifen. Um digitale Kompetenzen bei allen Studierenden der Humanmedizin zu fördern, ist eine Integration der Themen ins Pflichtcurriculum notwendig.

## Abstract (en)

**Background:**

Digital medicine has become increasingly important, especially with the growing use of artificial intelligence (AI) and telemedicine. The COVID 19 pandemic has accelerated this trend and emphasized the need for digital competencies in healthcare. However, digital competencies have been insufficiently taught in medical school to date.

**Objective:**

The introduction of an elective course in digital competencies in medicine fills a curricular gap at the Medical Faculty of the University of Freiburg.

**Methods:**

The elective course was developed by an interdisciplinary working group and includes seven modules on topics such as e‑health, telemedicine, AI, hospital information systems, and data literacy. Each module consists of a preparatory self-study unit, a theoretical part, and a practical part. An evaluation was conducted to assess the effectiveness of the elective.

**Results:**

Six students participated in the elective course in the summer semester of 2022. The evaluation shows that the goals of the course were achieved. The participants were able to acquire theoretical knowledge and deepen it through practical exercises.

**Conclusion:**

The interdisciplinary design of the course promotes exchange between students and teachers and creates synergies. The modular format of the course also allows for current topics, such as generative AI, to be addressed. In order to promote digital competencies among all students of human medicine, it is necessary to integrate the topics into the mandatory curriculum.

Digitale Kompetenzen in der Medizin gewinnen zunehmend an Bedeutung. Um dieses wichtige Thema im Medizinstudium zu verankern, hat die Medizinische Fakultät Freiburg ein klinisches Wahlpflichtfach eingeführt.

## Digitalisierung in der Medizin

Digitale Gesundheit umfasst ein breites Spektrum an digitalen Technologien, die im Gesundheitssektor Anwendung finden [13]. Hierzu gehören beispielsweise elektronische Patientendaten, Telemedizin sowie der Einsatz von künstlicher Intelligenz (KI). Insbesondere KI wird immer intensiver genutzt, beispielsweise in der Bilderkennung. Die COVID-19-Pandemie hat den Weg zur Digitalisierung beschleunigt, gerade Telemedizin wurde zu einem wichtigen Baustein der Patientenversorgung [9]. Der Digitalisierungstrend, welcher im Alltag immer stärker gelebt wird, muss sich auch im Medizinstudium widerspiegeln, um die Ärzt*innen von morgen auf die Herausforderungen im Klinikalltag und in der ambulanten Versorgung vorzubereiten.

In einer aktuellen Übersichtsarbeit haben Khurana et al. [6] über 40 digitale Kompetenzen für Medizinstudierende identifiziert und als relevant eingestuft. Im Medizinstudium werden Themen der Digitalisierung des Gesundheitswesens allerdings bisher kaum adressiert [1, 3, 11]. Im Nationalen Kompetenzbasierten Lernzielkatalog Medizin (NKLM 2.0, [10]) sind hierzu einige Lernziele definiert, die im Zuge der anstehenden Novellierung der Ärztlichen Approbationsordnung zusätzlich an Bedeutung gewinnen werden [12].

Das Wahlfach „Digitale Kompetenzen in der Medizin“ ist der erste Baustein für eine systematische Verankerung dieser wichtigen Thematik im Freiburger Curriculum. Das Konzept verknüpft in Bezug auf digitale Kompetenzen in der Medizin Theorie und Anwendung: Studierende erhalten zunächst von Expert*innen einen Einblick in den Klinikalltag und die gelebten Anforderungen bezüglich digitaler Kompetenzen. Durch praktische Übungen sammeln sie außerdem Erfahrungen, um eventuelle Ängste bezüglich digitaler Anwendungen niedrigschwellig abzubauen. Die Umsetzung des Wahlfachs wird ausführlich evaluiert, um Schlüsse für seine Weiterentwicklung zu ziehen sowie die Integration der wichtigsten Themen rund um Digitalisierung ins Pflichtcurriculum des Medizinstudiums zu fördern.

An der Medizinischen Fakultät der Universität Freiburg gibt es bereits seit 2017 Inselveranstaltungen [11], doch eine longitudinale, curriculare Verankerung von digitalen Kompetenzen in der Medizin existiert noch nicht. Um den digitalen Anforderungen im Gesundheitsbereich gerecht zu werden, bedarf es einer curricularen Erweiterung des Humanmedizinstudiums zu digitalen Kompetenzen.

## Ziel

Durch die Etablierung eines neuen Wahlfachs für Digitale Kompetenzen in der Medizin setzen sich Studierende mit Themen wie Data Literacy, Telemedizin, KI und Krankenhausinformationssysteme kritisch auseinander. Durch den hohen Praxisanteil wird der Erwerb von Anwendungswissen und -kompetenzen gefördert [4]. Die Umsetzung der Praxisteile erfolgt in Kleingruppen: Dies fördert die Aktivität der einzelnen Studierenden, und Technologieängste können abgebaut werden.

Bei der quantitativen und qualitativen Evaluation wird ermittelt, wie die Studierenden ihren eigenen Kompetenzzuwachs durch die Veranstaltung einschätzen. Außerdem wird ermittelt, wie die Veranstaltung inhaltlich und organisatorisch optimiert werden kann.

## Methoden

Das Wahlfach wurde von einer 2020 gegründeten Arbeitsgemeinschaft für „Digitale (Arzt‑)Kompetenzen“ an der Medizinischen Fakultät der Universität Freiburg für den klinischen Abschnitt des Humanmedizinstudiums konzipiert. Die interdisziplinäre Arbeitsgemeinschaft setzt sich zusammen aus Lehrenden aus den Bereichen Hals-Nasen-Ohren-Heilkunde, Allgemeinmedizin, Radiologie, Neurochirurgie, Herz- und Gefäßchirurgie, Digitalisierung in der Medizin sowie einem studentischen Mitglied und einem Patientenvertreter.

Von der Arbeitsgemeinschaft wurden folgende Bereiche zum Thema digitale Medizin identifiziert, welche im Humanmedizinstudium abgedeckt werden sollen: E‑Health, Telemedizin, KI, Krankenhausinformationssysteme sowie Data Literacy. Die Themen werden in 7 verschiedenen Modulen abgebildet:

1. Sonographie mit Augmented Reality und rechtliche Aspekte
  - *Theorie:* Datenschutz, Einführung in die Sonographie mit Handheld-Ultraschallgeräten
  - *Praxis:* Übungen mit Point-of-Care-Ultraschall (POCUS)
2. Klinische Dokumentationsmodelle und Datenfluss
  - *Theorie:* medizinisches Informationsmanagement, Visualisierungsverfahren
  - *Praxis:* Übungen im Krankenhausinformationssystem (KIS)
3. Ethische Aspekte von Digitalisierung, Robotik und KI
  - *Theorie:* ethische Prinzipien der Mensch-Technik-Interaktion, Cybersecurity
  - *Praxis:* Bilderkennung mit künstlichen neuronalen Netzen
4. Möglichkeiten und Grenzen von KI
  - *Theorie:* Hintergründe und Fehlerquellen, CAD-Systeme (computerassistierte Diagnostik)
  - *Praxis:* KI-Training für Röntgenbildklassifikation
5. Telemedizin
  - *Theorie:* Telematik-Infrastruktur, Aspekte der Online-Arzt-Patientenkommunikation, Voraussetzungen für die Telekonsultation
  - *Praxis:* fallbasiertes Telekommunikationstraining
6. E‑Health und Gesundheits-Apps
  - *Theorie:* Überblick Gesundheits-Apps in Deutschland, Patientensicherheit, App-Bewertung (Nutzen und Anwendung)
  - *Praxis:* Mitentwicklung einer Präventions-App
7. Digital und Data Literacy
  - *asynchrone Selbstlerneinheit* mit folgenden Materialien: KI-Campus: Stadt | Land | DatenFluss Handlungsfeld Gesundheit [7], Videos und Artikel [2, 5, 8], Selbstlern-Quiz

Für die Lehrveranstaltung wird ein Blended-Learning-Format mit folgendem Aufbau für Module 1–6 gewählt:

1. vorbereitende Selbstlerneinheit (30 min, online, asynchron),
2. Theorieteil (1 h, online, synchron),
3. Praxisteil (1,5 h, Präsenz/online, synchron).

Modul 7 steht ausschließlich als asynchrone Selbstlerneinheit zur Verfügung. Ergänzt werden die Module durch eine Einführungsveranstaltung (1 h, online, synchron) sowie eine Abschlussveranstaltung (2 h, Präsenz). In der Abschlussveranstaltung präsentieren die Studierenden die Ergebnisse ihrer Projektarbeit, die sie je nach individuellem Interesse zu einem der Modulthemen durchgeführt haben. Die begleitenden Kursmaterialien werden den Studierenden im virtuellen Kursraum auf der Lernplattform ILIAS (ILIAS open source e-Learning e. V., Köln, Deutschland) zur Verfügung gestellt.

**Figure 1:**
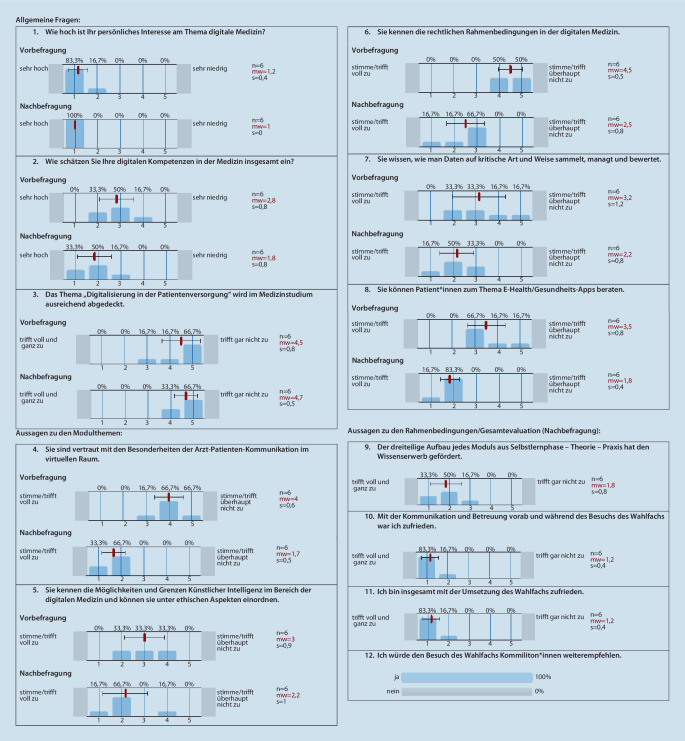

Die erste Durchführung des Wahlfachs im Sommersemester 2022 wurde ausführlich evaluiert: Für Studierende gab es eine Vorbefragung zu Interessen und Erwartungen, eine Evaluation für Module 1–6 sowie eine Abschlussevaluation zum Wahlfach insgesamt (Abb. 1). Lehrende wurden ebenfalls im Anschluss an ihr Modul zu diesem befragt, um organisatorisches und inhaltliches Verbesserungspotenzial zu ermitteln.

## Ergebnisse und Diskussion

Im Sommersemester 2022 nahmen 6 Studierende am Wahlfach Digitale Kompetenzen in der Medizin teil. In Abb. 1 sind Ergebnisse der Befragungen dargestellt, aufgrund der niedrigen Stichprobengröße ausschließlich im deskriptiven Format.

Die Ziele des Wahlfachs wurden erreicht: Die Teilnehmer*innen konnten die Themen aus theoretischer Sicht beleuchten und mit praktischen Übungen zu jedem Thema vertiefen. Die Studierenden wurden so motiviert, sich mit dem Thema digitale Medizin auseinanderzusetzen, und konnten im Rahmen einer Abschlussarbeit ihre thematischen Schwerpunkte mit den Lehrenden diskutieren. Die Durchführung des Wahlfachs hat sowohl den Austausch zwischen Studierenden und Lehrenden auf Augenhöhe gefördert als auch den Austausch der Lehrenden untereinander, sodass Synergien geschaffen werden konnten.

Für die einzelnen Module zeigt sich, dass die Studierenden ihre Kompetenzen nach dem Besuch der Veranstaltung als wesentlich besser einschätzen. Das Interesse am Thema digitale Medizin bleibt konstant hoch. Die Studierenden bleiben auch nach dem Besuch der Veranstaltung bei ihrer Ansicht, das Thema digitale Medizin werde im Studium nicht ausreichend behandelt. Die Studierenden sind sehr zufrieden mit der Umsetzung des Wahlfachs insgesamt und empfehlen den Besuch zu 100 % weiter.

Das Wahlfach wurde seit Sommersemester 2022 jedes Semester angeboten und teilweise inhaltlich leicht variiert. Das modulare Wahlfachformat ermöglicht große inhaltliche Flexibilität: So war es beispielsweise möglich, schon im Sommersemester 2023 das hochaktuelle Thema generative KI am Beispiel von ChatGPT (OpenAI, San Francisco, CA, USA) zu integrieren.

Aufgrund der niedrigen Teilnehmerzahlen an der Veranstaltung bzw. an der Evaluation in den Folgesemestern können keine weiteren deskriptiven Daten vorgelegt werden. Die niedrigen Teilnehmerzahlen sind laut den Studierenden nicht auf mangelndes Interesse an dem Thema, sondern auf widrige Rahmenbedingungen wie beispielsweise Überschneidung mit weiteren (Pflicht‑)Veranstaltungen und Limitationen in der Anrechenbarkeit zurückzuführen. Die positiven mündlichen Rückmeldungen der Studierenden und Lehrenden unterstützen jedoch die weitere Durchführung.

## Fazit für die Praxis

- Ein dreiteiliger Modulaufbau (Selbstlernphase, Theorie und Praxis) fördert den Kompetenzerwerb im Bereich der digitalen Medizin bei Studierenden.
- Durch die vorbereitenden Phasen kann die Präsenzzeit effizient für praktische Übungen genutzt werden.
- Die interdisziplinäre Planung und Umsetzung führt zu Synergie-Effekten unter den Lehrenden, und die Studierenden profitieren vom Perspektiven-Pluralismus.
- Das Thema digitale Medizin muss auch im Pflichtcurriculum verankert werden, um die breite Masse der Medizinstudierenden zu erreichen.
